# Pinned domain wall oscillator as a tuneable direct current spin wave emitter

**DOI:** 10.1038/s41598-017-13806-1

**Published:** 2017-10-19

**Authors:** Michele Voto, Luis Lopez-Diaz, Eduardo Martinez

**Affiliations:** Departamento de Física Aplicada, Universidad de Salamanca, Plaza de la Merced s/n, 37008 Salamanca, Spain

## Abstract

Local perturbations in the relative orientation of the magnetic moments in a continuous magnetic system can propagate in the form of waves. These so-called spin waves represent a promising candidate as an information carrier for spin-based low-power applications. A localized, energy-efficient excitation of coherent and short-wavelength spin waves is a crucial technological requirement, and alternatives to excitation via the Oersted field of an alternating current must be explored. Here, we show how a domain wall pinned at a geometrical constriction in a perpendicularly magnetized thin nanowire emits spin waves when forced to rotate by the application of a low direct current flowing along the wire. Spin waves are excited by the in-plane stray field of the rotating domain wall and propagate at an odd harmonic of the domain wall rotation frequency in the direction of the electron’s flow. The application of an external field, opposing domain wall depinning induced by the current, breaks the symmetry for spin wave propagation in the two domains, allowing emission in both directions but at different frequencies. The results presented define a new approach to manufacture tuneable high-frequency spin wave emitters of easy fabrication and low power consumption.

## Introduction

A spin wave (SW) is a propagating perturbation in the spin texture of a magnetic material in the form of a phase-coherent precession of the magnetic moments^1–3^. The quanta of spin waves are called magnons and the field investigating the transmission and processing of information mediated by spin waves is termed magnonics. Magnonics offers a promising new route for computing technology because it may overcome the limitations of complementary metal oxide semiconductor (CMOS) technology in terms of scalability and power consumption via a particle-less transmission of information^4–9^ and by introducing new degrees of freedom encoded in spin waves’ transport of angular momentum. Spin waves have short wavelengths at the technologically relevant GHz - low THz frequencies, allowing for integration with microwave electronics at the nanoscale^2^. The classical technique used to excite spin waves is via the Oersted field induced around a wire placed on top of the spin waves conduct from an ac current flowing through it^6,8,10^. This approach allows control of the frequency and wavelength of injected magnons, with the main drawback that the antenna width sets a lower bound for wavelength and limits the scalability of the device. An alternative way of inducing linear excitation of short-wavelength spin waves is through the uniform microwave excitation of a inhomogeneous magnetization texture^11,12^. The conversion of electron-carried angular momentum into magnons^13–15^ and vice versa^10,16^ allows for the exploitation of spintronics phenomena for the generation and detection of spin waves at the nanoscale and the embedding of magnonic circuitry in electronic-based devices. This novel field is called magnon-spintronics. Here, spin wave generation can be achieved by various localized excitations, such as electric field control of the magnetostrictive properties of materials^17–19^ and spin transfer torque^20–22^ (STT), generated either by a spin-polarized current flowing through a nanocontact^13,14^ or via the spin current originated by the flow of charge current through an adjacent non-magnetic metal with large spin-orbit coupling^15,23–25^.

It is known that a domain wall (DW) can emit spin waves during its motion^26–30^ or under microwave linear excitation^12^; the use of an oscillating domain wall as a tuneable spin wave emitter excited by an alternate current, has been proposed by Van de Wiele and colleagues^31^. In their work, a strong pinning of the DW is achieved via ferromagnetic-ferroelectric coupling, and an ac current is used to generate DW oscillations that excite propagation of SWs in adjacent domains at an angle of 45° with respect to the magnetization orientation. The use of a DW permits SW excitation at wavelengths much shorter than can be achieved with common antennas and the change in ac frequency can, to some extent, regulate the emitted frequency. However, the realization of such a device presents some limitations: high current densities are required, the fabrication of hybrid ferromagnetic-ferroelectric structures is difficult, and the propagation of SWs in the 45° magnetized domains is non-trivial.

It was shown both analytically and numerically^32–35^ that a DW pinned at a constriction in a perpendicularly magnetized nanowire could be led to self-sustained full in-plane rotation by the STT exerted on it by the injection of a low in-plane dc current while remaining pinned at a localized pinning site, thus creating a DW-based oscillator with frequencies in the GHz range, tuneable via the applied current intensity.

**Figure 1:**
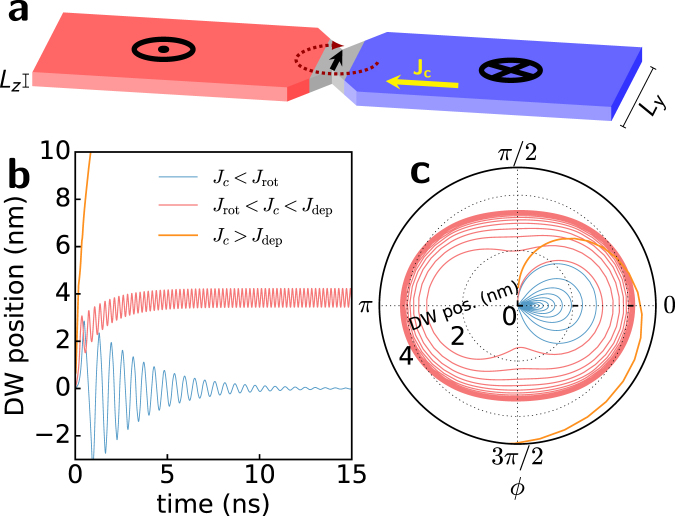
Scheme of the system under study and DW dynamics representation. (**a**) A DW located at a symmetric notch separates the up (red) and down (blue) domains. Current flows from right to left so that the induced rotation of the DW is clockwise. The in-plane direction of rotation is marked. (**b)** DW position as a function of time in micromagnetic simulations with current values below (dark blue line), inside (purple line) and above (orange line) the pinned rotation window. (**c**) Data from the same simulations as in (**b**) plotted to represent DW position *q* as the radial coordinate and the DW angle *ϕ* as the polar coordinate.

In this work we reconsider the DW oscillator set-up, schematically represented in Fig. 1a, and investigate, using micromagnetic simulations, the emission of SWs generated by such localized magnetization precession in a nanowire. By selecting an adequate wire width and constriction geometry, we can achieve a wide operating window in which we observe DW rotation at a current-dependent frequency (*f*_*DW*_) that leads to unidirectional emission of SWs in the direction of electrons’ flow at odd harmonics of *f*_*DW*_. Because the SW frequency is a multiple of *f*_*DW*_, the frequency of the emitted SWs can also be tuned by changing current intensity. Moreover, through the application of an external field opposing the force exerted on the DW by the current, the device operating window is extended and, at the same time, the symmetric dispersion relation for SWs in the two antiparallel domains is naturally split, which allows us to selectively propagate different harmonics along each domain. We identify the DW’s in-plane stray field as the main factor responsible for SW excitation, whereas the unidirectionality is due to the asymmetric position of the DW below the geometrical constriction. This new concept of SW emitter has the attractive features of high coherence, a tuneable frequency up to tens of GHz and low power consumption (typical current of a few *μ*A) by simply exploiting the stray field induced by geometrical patterning.

## Results

We consider a DW trapped at a symmetric constriction in a narrow wire and a dc current flowing through it, as shown in Fig. 1a. The constriction acts as a pinning site for the DW and therefore, a minimum threshold current *J*_dep_ is required to depin the DW and propagate it through the nanowire. For current densities below this threshold, the DW remains pinned at a position where the restoring pinning force balances the driving STT force that pushes the DW away from the notch. However, a zero net driving force does not imply balance of the in-plane torques acting on the DW. In particular, if a current density is above a certain value *J*_rot_, the in-plane component of the STT overcomes the shape anisotropy field torque^36^, leading to sustained full in-plane rotation of the spins inside the DW^33,34^. Such a situation requires the fulfilment of precise conditions that we synthetically present below using a typical one-dimensional model^37–39^ (see Methods), which provides a good approximation to complex DW dynamics in narrow wires. Within this model, the system is described using the position of the DW (*q*) with respect to its equilibrium position centred at the constriction and the in-plane orientation (*ϕ*) of the spins in the DW as the only degrees of freedom. The pinning caused by the constriction is modelled as a parabolic potential well^34^, which gives rise to a spring-like restoring field *H*_*p*_(*q*). Within this framework, pinned DW rotation is attained for current density values *J*_*c*_ in the range

1 $${J}_{{\rm{rot}}}=\frac{e{M}_{s}}{P{\mu }_{B}}\frac{{\gamma }_{0}{\rm{\Delta }}{H}_{K}}{2} < {J}_{c} < \frac{e{M}_{s}}{P{\mu }_{B}}\frac{\alpha {\gamma }_{0}{\rm{\Delta }}{H}_{p}^{max}}{1+\alpha \beta }={J}_{{\rm{dep}}},$$

where *e* is the negative electron charge, *M*_*s*_ is the saturation magnetization, *P* the current polarization, *μ*_B_ is Bohr magneton, ${\gamma }_{0}=2.21\times {10}^{5}\,{\rm{rad}}\,{\rm{m}}\,{{\rm{A}}}^{-{\rm{1}}}{{\rm{s}}}^{-{\rm{1}}}$ is the gyromagnetic ratio, ${\rm{\Delta }}$ the domain wall width parameter, ${H}_{K}$ the in-plane shape anisotropy field, *H* ^*max*^_*p*_ the maximum pinning field, α is Gilbert’s damping parameter and *β* the degree of non-adiabaticity. The analytical derivation of such conditions can be found in section A of the supplementary information.

Data from micromagnetic simulations realized for three different cases, ${J}_{c\mathrm{,1}} < {J}_{{\rm{rot}}} < {J}_{c\mathrm{,2}} < {J}_{{\rm{dep}}} < {J}_{c\mathrm{,3}}$ are shown in Fig. 1b, where the DW position is plotted as a function of time, and in Fig. 1c, where the position and in-plane DW angle are shown in polar coordinates. As can be observed, the DW reaches an equilibrium position for *J*_*c*_ < *J*_rot_ after a few nanoseconds, whereas for ${J}_{c} > {J}_{{\rm{dep}}}$, the DW rapidly depins from the notch. For *J*_rot_ < *J*_*c*_ < *J*_dep_, however, the DW moves a few nanometres towards the right and slightly oscillates back and forth around this position while rotating in-plane.

To obtain a large operating window of the device, we tune the wire width and notch shape to obtain a low threshold current for the DW pinned rotation *J*_rot_ and a high threshold current for DW depinning *J*_dep_. Because the $|{{\bf{J}}}_{c}|$ value is not constant in space and increases at the constriction, throughout the paper, we refer to its value as the nominal one away from the geometrical constriction. We select a wire width *L*_*y*_ of 60 nm, a thickness ${L}_{z}=1\,{\rm{nm}}$ and a notch depth of 20 nm, which gives us ${J}_{{\rm{rot}}}={10}^{10}\,{\rm{A}}\,{{\rm{m}}}^{-{\rm{2}}}$ and ${J}_{{\rm{dep}}}=12.75\times {10}^{10}\,{\rm{A}}\,{{\rm{m}}}^{-{\rm{2}}}$, with the latter corresponding to a maximum current intensity of 7.6 *μ*A in the nanowire. The working window of such a device has the desirable quality of lying in a low-current density range, which allows us to avoid Joule heating effects and significant temperature gradients in proximity of the constriction^40^.

### Spin Wave Emission

**Figure 2:**
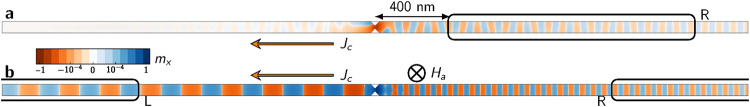
Snapshot of the magnetization dynamics representing the ${m}_{x}={M}_{x}/{M}_{s}$ value. (**a)** A current density of $6.5\times {10}^{10}\,{\rm{A}}\,{{\rm{m}}}^{-{\rm{2}}}$ is injected. The region $R$ on the right where *m*_*x*_ is sampled is enclosed by a rectangle. Unidirectional SW propagation towards the right can be observed. (**b)** Snapshot of the magnetization dynamics under the concurrent action of an in-plane current of $24\times {10}^{10}\,{\rm{A}}\,{{\rm{m}}}^{-{\rm{2}}}$ and an external field of 300 mT directed inside the plane to oppose DW depinning. A wire twice as long as in (**a)** is considered. Emission of SWs is observed both towards the right and the left at different frequencies and wavelengths; the sampled regions on the left (*L*) and right (*R*) are enclosed by black rectangles, and they extend for 2 *μ*m.

**Figure 3:**
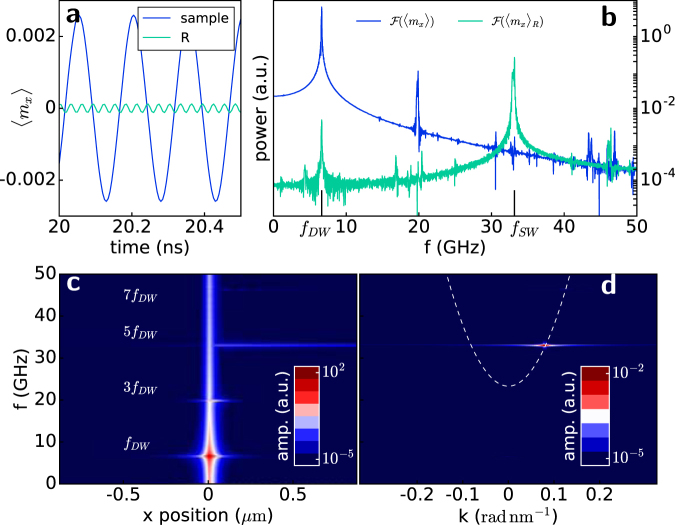
Study of DW rotation frequency and SW emission frequency. (**a**) Evolution of the averaged $x-$ component of the normalized magnetization during a time window of $0.5\,{\rm{ns}}$. The dark blue line shows averaging over the whole sample and the light blue line shows the averaging over the region $R$ away from the DW. Different periodicities can be observed. (**b)** Fourier transform of the time signals shown in (**a)** the dark blue line shows the main peak at the frequency of rotation of the DW *f*_*DW*_, and the light blue line has the principal peak at the SW propagation frequency ${f}_{SW}=5{f}_{DW}$. (**c**) Frequency spectrum of *m*_*x*_(*t*) as a function of *x*− position at the center of the strip width. Peaks centred at the DW position with an odd multiple frequency of *f*_*DW*_ are marked. (**d)** $f-k$ diagram extracted from the central line in region *R* shows a focused peak lying over the right principal branch of the analytical dispersion relation, marked as a dashed line.

Upon the application of a current ${J}_{{\rm{rot}}} < {J}_{c} < {J}_{{\rm{dep}}}$ through the wire, the domain wall is driven towards the right and, after a transient time of a few ns (see Fig. 1b), reaches a stationary position below the notch, where it slightly oscillates back and forth (see Fig. 1b) while its spins rotate clockwise in the strip plane, as shown in Fig. 1c and in movie A of the supplementary material. Because of the reduced lateral dimension of the wire, DW rotation is coherent and its spins rotate synchronously. Examining the normalized *x*- component of magnetization ${m}_{x}={M}_{x}/{M}_{s}$, as shown in Fig. 2a and in movie A in the supplementary material for ${J}_{c}=6.5\times {10}^{10}\,{\rm{A}}\,{{\rm{m}}}^{-{\rm{2}}}$, we observe the presence of the characteristic pattern of SWs propagating to the right side of the strip, whereas a much weaker propagation is observed on the left side. In Fig. 3a we monitored the value of the *x*- component of magnetization averaged over the whole strip, $\langle {m}_{x}\rangle$ (dark blue line) and observed its value oscillate around zero at the frequency ${f}_{DW}=6.6\,{\rm{GHz}}$. If we examine the average over a 1.35 *μ*m long region *R*, located 400 nm from the DW (light green line), we observe a smaller oscillation at higher frequency. By taking the Fourier transform of these two signals (Fig. 3b), we observe a peak at ${f}_{DW}=6.6\,{\rm{GHz}}$, while the main signal from the region *R* represents the SW frequency ${f}_{SW}=33\,{\rm{GHz}}$. The secondary peak in the global signal, corresponding to 3*f*_*DW*_, represents an odd higher harmonic. To gain more insight into the magnetization dynamics, we examine the frequency signal distribution over space (Fig. 3c) by taking the Fourier transform of ${m}_{x}(t,{\bf{r}})$ at every cell situated along the *x*- central axis of the strip. A large amplitude can be observed at the centre of the strip where the DW rotates, remaining pinned below the notch, with the largest amplitude at the frequency *f*_*DW*_, at which the DW rotates fully in-plane. Additional peaks at odd multiples of *f*_*DW*_ can be seen with a propagating branch corresponding to the fifth harmonic, indicating a definite propagation of the SW towards the right. Taking the Fourier transform in space and time of ${m}_{x}(t,{\bf{r}})$ in the same central row of cells restricted to region *R*, we obtain the *f* *−* *k* diagram showing a single focused spot corresponding to *f*_*sw*_ (Fig. 3-d). The analytical dispersion relation for exchange spin waves in our sample is also shown in the figure:

2 $$\omega ({\bf{k}})={\omega }_{0}+{\omega }_{M}{\lambda }_{{\rm{ex}}}^{2}{{\bf{k}}}^{2},$$

where ${\omega }_{0}={\gamma }_{0}({H}_{k,\mathrm{eff}}+{H}_{a})$, *H*_*k,eff*_ is the effective out-of-plane anisotropy, ${\omega }_{M}={\gamma }_{0}{M}_{s}$ and ${\lambda }_{{\rm{ex}}}^{2}=\frac{2A}{{\mu }_{0}{M}_{s}^{2}}$. Indeed, ${f}_{SW}=5{f}_{DW}=33\,{\rm{GHz}}$ is the first odd harmonic that is allowed to propagate in the system, being above the threshold frequency ${f}_{0}={\omega }_{0}\mathrm{/2}\pi =23.3\,{\rm{GHz}}$. This emission of SWs has the remarkable property of being unidirectional, coherent and directly dependent on the applied current density as we will discuss below.

**Figure 4:**
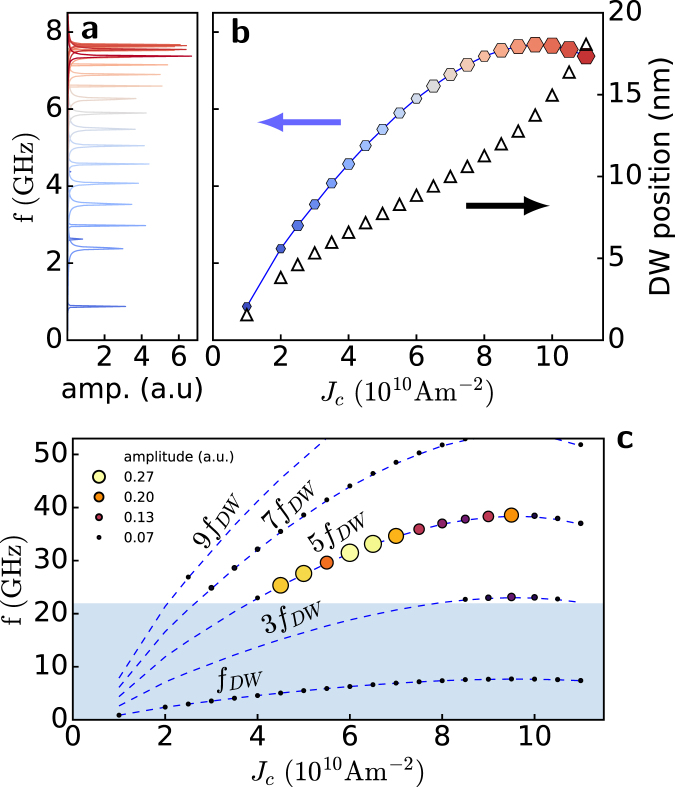
Frequency study of DW rotation and SW emission. (**a)** Frequency spectra of $\langle {m}_{x}\rangle$ showing the peaks from which *f*_*DW*_ is extracted. (**b)** DW frequency (full hexagons) as a function of applied current extracted from the peaks in (**a**) the size of the hexagons is proportional to the height of the peaks. Average DW position in the pinned rotation regime for the corresponding current (triangles). (**c)** Principal peaks in the frequency spectrum extracted from region *R* away from the DW.The peak amplitude is denoted by the circle size and the colour scale, (dark to bright). The dashed lines denote *f*_*DW*_ as in (**b**) and its odd multiples. The shaded region denotes the non-propagating frequency gap $f < {f}_{0}$.

Varying the applied current intensity between *J*_rot_ and *J*_dep_ leads to different DW rotation frequencies extracted from ${\mathcal F} (\langle {m}_{x}\rangle )$, as shown in Fig. 4a,b. It is predicted by the analytical model^32^ that a linear relationship exists between *f*_*DW*_ and the applied current. However, the DW rotation position varies with applied current, thus changing the local current density at the DW position; as a result, the DW rotation frequency is not linear with nominal applied current. The linear dependence between the applied current and *f*_*DW*_ is recovered if we consider the actual current density flowing at the DW position (see the supplementary information). In addition, the amplitude of the signal increases with current density, as denoted by the size of the hexagons in Fig. 4b. This observation is due to the fact that $\langle {m}_{x}\rangle$ oscillation increases with *J*_*c*_ because the DW moves further away from the centre of the notch and, therefore, the number of spins precessing also increases.

If we now examine the frequency spectrum away from the notch, we observe a different distribution of amplitude peaks with *J*_*c*_. In Fig. 4c, peaks in the frequency signal sampled over region *R* are plotted against the applied current density with different colours and sizes to mark their amplitude. Dashed blue lines denote the frequency-current density curve of the rotating DW and its odd higher harmonics. As can be observed, all peaks lie on odd harmonics of *f*_*DW*_, and their amplitude is maximum when SW can actually propagate, i.e., above the threshold frequency *f*_0_. The frequency gap region where propagation is forbidden is shaded in blue. The highest emission intensity is achieved for current densities between 4.5 and $7\times {10}^{10}\,{\rm{A}}\,{{\rm{m}}}^{-{\rm{2}}}$ on the fifth harmonic, which is the first branch largely above the propagation threshold *f*_0_. Emission is highly coherent with linewidths below 150 MHz.

### Application of external field

To extend the operating window of the device, we apply an external field opposing the driving force exerted by the STT. In our situation this means applying an external field *H*_*a*_ pointing into the plane along the $-\hat{{\bf{z}}}$ direction. We can estimate such an effect by means of the one-dimensional model: the external field together with the pinning effective field must balance the STT so that we have a linear dependence of the depinning current on the external field:

3 $${J}_{{\rm{dep}}}({H}_{a})=\frac{e{M}_{s}}{P{\mu }_{B}}\frac{\alpha {\gamma }_{0}{\rm{\Delta }}}{1+\alpha \beta }({H}_{p}(\overline{q})+{H}_{a})={J}_{{\rm{dep}}}^{0}+\frac{e{M}_{s}}{P{\mu }_{B}}\frac{\alpha {\gamma }_{0}{\rm{\Delta }}}{1+\alpha \beta }{H}_{a},$$

where *J* ^0^_dep_ is the threshold current for depinning without an applied field from equation (1).

**Figure 5:**
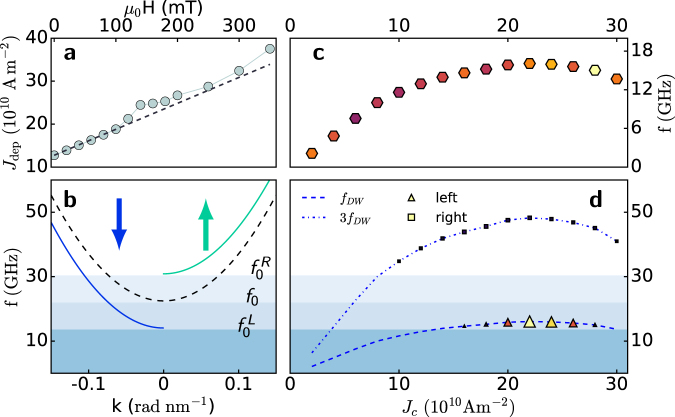
DW rotation and SW dynamics in the presence of an external field. (**a)** Depinning current ${J}_{{\rm{dep}}}$ as a function of a counter-acting external field applied along the $-\hat{{\bf{z}}}$ direction. The dashed line represents the theoretical prediction made using the one-dimensional model (3). (**b)** Splitting of the dispersion relation branches in the positive and negative directions with respect to the DW caused by the application of an external field, as prescribed by equation (2). (**c**) DW rotation frequency as a function of the applied current when an external field of 300 mT is applied. (**d**) SW propagation observed in the regions distant from the DW on the left (triangles) and on the right (squares), as indicated in Fig. 2b. The size of the symbols express the SW amplitude. The SW propagates to the left at *f*_*DW*_, whereas it propagate to the right at 3*f*_*DW*_.

The increase in the depinning current in the presence of an external field is shown in Fig. 5a together with the analytical prediction (3) (dashed line). Threshold current ${J}_{{\rm{dep}}}$ increases almost linearly with the applied field for a wide range of fields, with 120 mT giving a 100% increment of the depinning current at zero field. In this manner, we can extend the current density window for DW rotation and achieve *f*_*DW*_ up to 15.7 GHz as shown in Fig. 5c. An interesting consequence of the application of an into-the-plane field is its antisymmetric contribution to the effective field in the two magnetic domains in which our strip is divided, which leads to the vertical displacement of the left and right propagating branches (Fig. 5b), depending on the relative orientation with the magnetization.

This splitting of the dispersion relation in the left and right domains opens the possibility of SW propagation in the left domain. In fact, for an applied field of 300 mT, $f(k=\mathrm{0)}={f}_{0}=23.3\,{\rm{GHz}}$ becomes ${f}_{0}^{R}=31.4\,{\rm{GHz}}$ and ${f}_{0}^{L}=14.6\,{\rm{GHz}}$ for the right and left domains, respectively, with the latter being below *f*_*DW*_ for a wide range of *J*_*c*_ (see Fig. 5c,d). We perform simulations with an external applied field $-{H}_{a}\hat{{\bf{z}}}$ with ${\mu }_{0}{H}_{a}=300\,{\rm{mT}}$ and we monitor the magnetization in two regions *L* and *R*, both of them 2 *μ*m long and situated 1.296 *μ*m from the centre of the strip, as shown in Fig. 2b. The DW rotation frequency is extracted as usual as ${\mathcal F} (\langle {m}_{x}\rangle )$ and is plotted in Fig. 5c. In Fig. 5d, the peaks in frequency of *m*_*x*_(*t*) in the two sampled regions are plotted, where the size of each symbol represents the peak’s amplitude. In the left domain (triangles), we have SW propagation towards the left at the DW rotation frequency when this exceeds the propagation threshold frequency *f*_0_^*L*^, and no higher harmonic excitation is observed. On the right side, the third harmonic is now accessible for SW propagation via the increased DW rotation frequency and is the one at which SWs propagate. This result adds an important feature to this spin wave emitter because spin wave propagation can be tuned in two different aspects: the propagation frequency can be regulated by changing the applied current, while bidirectional or unidirectional emission from the DW and additional frequency regulation can be obtained via the application of an external field.

## Discussion

This novel scheme for tuneable and short-wavelength SW emission suggests new directions in low-power magnonic devices. Unidirectional and asymmetric spin wave propagation is a peculiar feature of this system. The intrinsically asymmetric character of the Dzyaloshinskii-Moriya interaction (DMI) has been exploited to obtain unidirectional propagation of SWs along nanowires^41^ and focusing of SWs in thin films^42^. However, in our system, the effect of DMI is negligible, and the origin of such an effect is purely geometric, as will be shown below. The precession of the domain wall’s spins is the source of spin wave excitation, and SW propagation at odd higher harmonic of the DW rotation frequency is the signature of a periodic and non-linear excitation^30,43^. If the simple oscillation of the DW below the notch or its change in width when passing from Néel to Bloch configuration were the main mechanism of excitation, we would observe emission at 2*f*_*DW*_ and its harmonics. However, the absence in the frequency spectrum of the amplitude peaks at even multiples of *f*_*DW*_ makes us disregard this hypothesis.

**Figure 6:**
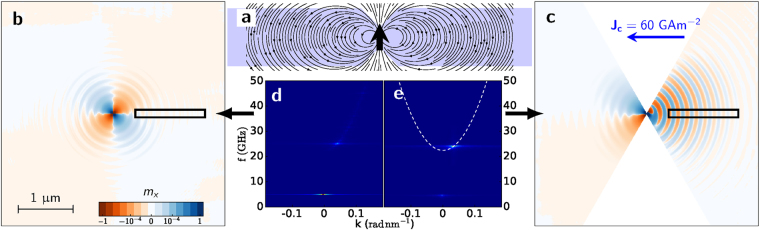
Comparison of the SW excitation caused by a rotating dipolar field and a pinned rotating DW. (**a)** Schematic spatial configuration of the in-plane stray field generated by the DW. (**b)** Snapshot of the magnetization dynamics in a thin film where the excitation is produced by a dipolar field located at the film centre rotating at $5\,{\rm{GHz}}$. (**c)** Snapshot from simulations, where an applied current induces rotation of a DW pinned at the centre. (**d)** $f-k$ diagram extracted from the region indicated in (**b)**, showing the non-propagating oscillation at 5 GHz and the propagating one at 25 GHz. (**e)** *f* − *k* diagram showing propagation of SWs in the region indicated by the rectangle in (**c**).

To shed more light on the excitation mechanism, we focus our attention on the role played by the stray field of the rotating DW. Because of the reduced width of the strip, precession of the spins in the DW occurs in a very coherent fashion, making the DW appear as a dipole rotating in the strip plane, as represented schematically in Fig. 6a. The stray field generated by such a dipole has a strong in-plane component, and it rotates at $f={f}_{DW}$. To verify that the DW behaves as a rotating dipole and can be regarded as the main mechanism that excites propagating SWs, we proceed in two steps. First, we examine the SW emission induced in a uniformly magnetized thin film via the external field generated by a point dipole ${\bf{B}}({\bf{r}})=\frac{{\mu }_{0}}{4\pi }(\tfrac{3{\bf{r}}({\bf{m}}\cdot {\bf{r}})}{{r}^{5}}-\tfrac{{\bf{m}}}{{r}^{3}})$ rotating in-plane at $f=5\,{\rm{GHz}}$. After a period of transient turbulent dynamics with incoherent emission of SWs, when a stationary regime is reached, we observe an isotropic and rather weak emission of spin waves in all directions (Fig. 6b and movie B of the supplementary information). When 2-dimensional Fourier transform is performed on m_*x*_(*t*) along a 1.25 *μ*m long line starting 400 nm from the centre of the square, we see in the *f*−*k* diagram a spot at 5 GHz and $k=0$ corresponding to the non-propagating oscillation induced directly by the external dipolar field. The principal branch of the propagating SW is also marked, showing a peak at 25 GHz; i.e., emission is stronger at a frequency 5 times larger than the driving rotation rate. In other words, the rotating dipolar field is responsible for excitation of SWs at odd harmonics and such excitation is weak, comparable to the one also observed in the left domain in Fig. 2a. In order to highlight the analogy with the case of the rotating DW, we carve a very deep symmetric notch in the square film to obtain a 20-nm channel in the middle as in the nano wires under examination. We then set an up-down magnetization configuration with the DW pinned at the channel and apply a current of $6\,{\rm{GA}}\,{{\rm{m}}}^{-{\rm{2}}}$ (uniform for simplicity) that yields a DW rotation frequency ${f}_{DW}\sim 4.8\,{\rm{GHz}}$. In Fig. 6c, a snapshot representing *m*_*x*_ during the stationary dynamics shows a strong SW emission to the right, whereas the perturbation in the left domain is much weaker and is not capable of exciting SWs, as can also be observed in movie B from the supplementary material. Extracting the *f* − *k* diagram from the same spatial region and over the same time span as in the rotating dipole case (Fig. 6e), we find a spot at ${f}_{DW}$ and $k=0$, while the spot on the dispersion relation branch is exactly at $24\,{\rm{GHz}}=5{f}_{DW}$ with no additional SW emission along the branch.

**Figure 7:**
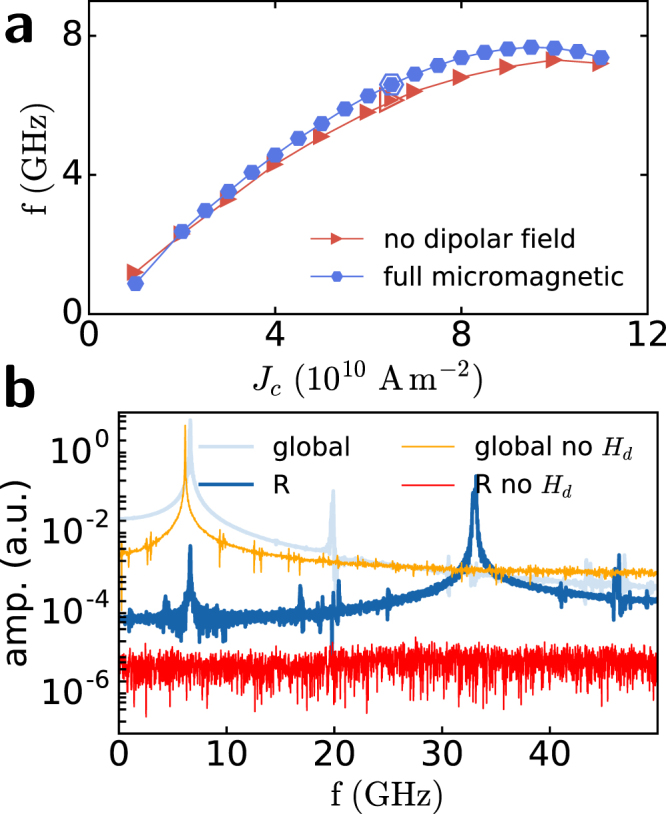
Role of non-local stray field in exciting SWs. (**a**) DW rotation frequency as a function of current for full micromagnetic simulations (blue hexagons) compared with simulations without the non-local effect of the magnetostatic field (orange triangles). (**b**) Fourier transform of ${m}_{x}(t)$ averaged over the whole sample and in the region *R* 400 nm from the notch, as in Fig. 2a (light and dark blue lines), under the application of $6.5\times {10}^{10}\,{\rm{A}}\,{{\rm{m}}}^{-{\rm{2}}}$. Simulations without computation of the dipolar fields (orange and red lines) show a single peak at the DW frequency and no signal away from the DW.

**Figure 8:**
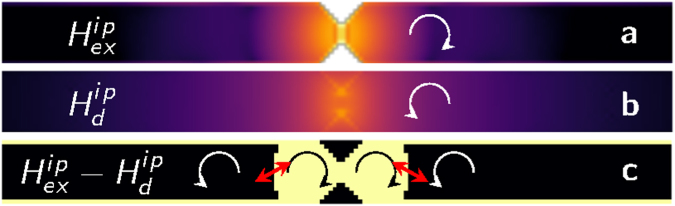
Competition between the in-plane components of exchange and dipolar field is the origin of SW excitation. The intensity of the in-plane component of the exchange field (**a**) and the dipolar field (**b**) for a DW placed at the center of the wire at rest is plotted in dark to bright colour scale. The arrow indicates the direction of rotation of the in-plane component of the field when the DW is led to rotation via an applied current. **c** The sign of ${H}_{ex}^{ip}-{H}_{d}^{ip}$ marked as bright (dark) for positive (negative). Arrows indicate the direction of rotation of the combined in-plane excitation.

To further prove the essential role of the DW’s stray field in exciting SWs, we run micromagnetic simulations without considering the long-range dipolar interaction; i.e., considering an anisotropy parameter ${k}_{{\rm{eff}}}={k}_{u}-\frac{1}{2}{\mu }_{0}{M}_{s}^{2}$ that includes the local demagnetizing effect of the dipolar field, we can achieve DW pinned rotation with a behaviour and a rotation frequency very similar to those observed in full simulations (Fig. 7a). However, only a weak dissipative radiation of exchange SWs is present^26,29^, and no coherent SW emission at a well defined frequency is observed, as shown in Fig. 7b, where the frequency spectrum at ${J}_{c}=6.5\times {10}^{10}\,{\rm{A}}\,{{\rm{m}}}^{-{\rm{2}}}$ is compared with the standard simulations that take into account dipolar interaction. From these observations, we can conclude that the dipolar field of the DW is responsible for the coherent excitation of SWs, behaving as a rotating antenna. However, additional contributions from the wire edges must be considered to explain the unidirectionality. When neglecting dipolar interaction, the DW rotation excites circular oscillations in the spins close to the DW only via exchange interaction passing from Bloch to Néel configuration at *f*_*DW*_ frequency. Such perturbation is very strong close to the DW and decays exponentially with distance. On the other hand the magnitude of the DW dipolar field decays as $|{\bf{r}}{|}^{-3}$ from the DW. In Fig. 8a,b, the magnitude $H=\sqrt{{H}_{x}^{2}+{H}_{y}^{2}}$ of the in-plane component of both exchange ${H}_{ex}^{{\rm{ip}}}$ and dipolar ${H}_{d}^{{\rm{ip}}}$ fields is shown in dark to bright colour scale before applying the external current, when the DW is pinned at the centre of the notch pointing upwards and no propagating SW perturbs the configuration. Moreover, when the DW is set into rotation by the application of a current, the in-plane component of the two fields rotates in opposite directions: clockwise ${H}_{ex}^{{\rm{ip}}}$ following the DW rotation and anticlockwise ${H}_{d}^{{\rm{ip}}}$, so there is a competition between the excitation of the spins due to the exchange interaction close to the DW and that due to dipolar interaction further away from the DW. Their combined effect results in a strongly elliptical excitation of the magnetization in the region where the two fields have a similar magnitude, as shown in Fig. 8c.

**Figure 9:**
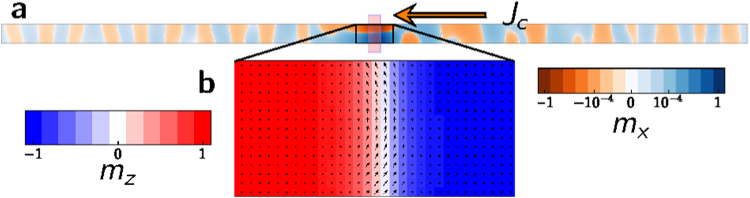
Non-geometrical constriction produces bidirectional SW emission. (**a)** Snapshot of the magnetization dynamics showing the $x-$ component of the magnetization when a DW is forced to rotate via the application of a current density ${J}_{c}=9\times {10}^{10}\,{\rm{A}}\,{{\rm{m}}}^{-{\rm{2}}}$. The pinning is realized via a decrease of 20% in uniaxial anisotropy constant ${k}_{u}$ in the shaded region. (**b)** Close view of DW mangetization distribution. The DW stretches across the whole wire width and its rotation is no longer uniform, causing a more irregular SW excitation and waveform.

At the wire edges, where in-plane tilt of the spins produces surface charges and an additional stray field component, this effect is strengthened. The fact that the DW is pushed by the STT from the centre of the notch towards the right makes the excitations at the edges much weaker on the left side, where both fields have small in-plane components so that their combined action is not capable of exciting higher harmonics. To prove this point, simulations with a different DW pinning strategy have been performed. A 20% lower uniaxial anisotropy constant *k*_*u*_ in a 30-nm wide band at the centre of the nanowire (indicated by the blue rectangle in Fig. 9a) creates an energetically favourable position for the DW, giving rise to a strong and localized potential well for the DW without changing the local geometry. Applying a current ${J}_{c}=9\times {10}^{10}\,{\rm{A}}\,{{\rm{m}}}^{-{\rm{2}}}$ produces SW emission in both directions (as shown in Fig. 9a). This result also proves that the small non-adiabatic torque we use does not play a role in suppressing spin waves that propagate against electron flow, as it has been argued in the literature^44^. Therefore, we conclude that the dipolar field of the rotating DW is responsible for the higher harmonic SW emission, and because this excitation has a dipolar and thus geometrical origin, the displacement of the DW on the right side of the pinning site causes the screening of the emission towards the left side. Such emission is recovered when ${f}_{DW} > {f}_{0}$, and the simple DW rotation can excite the SW towards the left (see Figs 2b and 6d).

In conclusion, we have presented a novel paradigm to excite spin waves via the spin transfer torque-induced rotation of a domain wall pinned at a geometrical constriction in a narrow wire. We showed that by selecting the notch shape and the wire cross section, spin wave emission in the direction of electrons’ flow can be achieved for extremely low current densities. Spin wave emission occurs at an odd multiple of the DW rotation frequency up to 40 GHz, without any external applied field. Such spin wave emission is very coherent and of short wavelength down to 60 nm, typical of exchange spin waves. The application of an external field opposing the STT has the twofold effect of extending the operation window of the DW pinned rotation regime on the one hand, achieving higher DW rotation frequencies, and affecting anti-symmetrically the dispersion relation in the two domains on the other hand, thus modulating the SW emission in the direction of electrons flow and allowing propagation in the opposite direction. In other words, the SW emitter can work as unidirectional or asymmetric bidirectional SW emitter depending on the application of an adequate external field. The dipolar field of the rotating DW is the main cause of periodic non-linear excitation of SWs that propagate at higher harmonics of the DW rotation frequency in the system. The displacement of the DW on one side of the notch enhances the excitation on one side and weakens it on the other, giving rise to the unidirectionality.

## Methods

### Micromagnetic modelling

In our study, we integrate numerically, using a custom finite difference solver, the Landau-Lifschitz-Gilbert (LLG) equation of the magnetization dynamics that includes the contribution of the spin transfer torque caused by the flowing of an in-plane charge current density **J**_*c*_^22^ with spin polarization *P* and degree of non-adiabaticity *β*:

4 $$\frac{\,{\rm{d}}{\bf{m}}}{\,{\rm{d}}t}=-{\gamma }_{0}{\bf{m}}\times {{\bf{H}}}_{{\rm{eff}}}+\alpha {\bf{m}}\times \frac{\,{\rm{d}}{\bf{m}}}{\,{\rm{d}}t}-({\bf{u}}\cdot \nabla ){\bf{m}}+\beta {\bf{m}}\times [({\bf{u}}\cdot \nabla ){\bf{m}}],$$

Here, *e* is the negative electron charge, *μ*_*β*_ is the Bohr magneton, ${\gamma }_{0}=2.21\times {10}^{5}\,{\rm{rad}}\,{\rm{m}}\,{{\rm{A}}}^{-{\rm{1}}}{{\rm{s}}}^{-{\rm{1}}}$ is the gyromagnetic ratio, *α* is Gilbert’s damping parameter and ${\bf{u}}\mathop{=}\limits^{{\rm{def}}}{{\bf{J}}}_{c}\frac{P{\mu }_{B}}{e{M}_{s}}$.

Material parameters of annealed 1 nm thick ${{\rm{Co}}}_{{\rm{20}}}{{\rm{Fe}}}_{{\rm{60}}}{{\rm{B}}}_{{\rm{20}}}$ as in^45^ were chosen: saturation magnetization ${M}_{s}=8.84\times {10}^{5}\,{\rm{A}}\,{{\rm{m}}}^{-{\rm{1}}}$, uniaxial anisotropy constant ${k}_{u}=8.35\times {10}^{5}\,{\rm{J}}\,{{\rm{m}}}^{-{\rm{3}}}$, exchange stiffness *A*_ex_ = 23 $\times {10}^{-12}\,{\rm{J}}\,{{\rm{m}}}^{-{\rm{1}}}$, Gilbert’s damping $\alpha =0.015$. The degree of non-adiabaticity of the spin transfer torque was chosen as $\beta =2\alpha$ and the polarization coefficient as $P=0.5$. The CoFeB strip under study is divided into square cells of 4 nm in side and 1 nm thick, with all dimensions below the Bloch length $\sqrt{A/{k}_{u}}=5.25\,{\rm{nm}}$. To avoid reflection of the SWs and simulate propagation in a much longer nanowire, absorbing boundary conditions are applied at the wire ends in the form of a smoothly augmented damping profile^46^. The spatial configuration of current density ${{\bf{J}}}_{c}$ is computed numerically by integrating Laplace’s equation. The LLG equation was integrated using a Runge-Kutta, Dormand-Prince predictor-corrector algorithm^47^ with embedded error control. Starting with a pinned Bloch DW configuration, simulations were run for 15 ns without saving output to skip the initial turbulent dynamics. Afterwards, simulations were run for 40 ns. The output was written every 5 ps.

### One dimensional model for pinned DW rotation

Domain wall dynamics in nanowires is well described by the so-called one-dimensional analytical model^37,38^ with the inclusion of the STT^22,39,48^. In its simplest form, the model takes into account the DW position *q* and the in-plane orientation *ϕ* of the DW spins. We make use of this model to derive the conditions that must be fulfilled to achieve DW pinned rotation. The two differential equations describing the dynamics of a DW moving along a wire with a geometrical constriction are^33,34^

5 $$\dot{q}=\frac{{\gamma }_{0}{\rm{\Delta }}}{1+{\alpha }^{2}}(\alpha ({H}_{p}(q)+{H}_{a})+\frac{{H}_{K}}{2}\,\sin \,2\varphi )+\frac{1+\alpha \beta }{1+{\alpha }^{2}}u,$$

6 $$\dot{\varphi }=\frac{{\gamma }_{0}}{1+{\alpha }^{2}}({H}_{p}(q)+{H}_{a}-\alpha \frac{{H}_{K}}{2}\,\sin \,2\varphi )+\frac{\beta -\alpha }{1+{\alpha }^{2}}\frac{u}{{\rm{\Delta }}},$$

where ${H}_{K}=2{K}_{\perp }/{\mu }_{0}{M}_{s}$ is the in-plane shape anisotropy field on the DW, ${\rm{\Delta }}$ is the DW width parameter, $u=|{\bf{u}}|$, *H*_*a*_ is an external field applied along $\hat{{\bf{z}}}$ and ${H}_{p}(q)=-{\mathrm{(2}{\mu }_{0}{M}_{s}{L}_{y}{L}_{z})}^{-1}\frac{\partial {V}_{p}(q)}{\partial q}$ represents the pinning field caused by a geometrical constriction at position $q=0$. We approximate the effect of the geometrical constriction as a parabolic potential well centred at the notch ($q=0$) with stiffness $k\,({\rm{J}}\,{{\rm{m}}}^{-{\rm{2}}})$ and width $\ell$

$${V}_{p}(q)=(\begin{array}{cc}\frac{k}{2}{q}^{2} & {\rm{if}}\,|q| < \ell \\ 0 & {\rm{else}}\end{array}\mathrm{.}$$

### Data availability

Data from micromagnetic simulations are available upon request from the corresponding author.

### Code availability

Micromagnetic custom code developed at Universidad de Salamanca can be made available from the corresponding author upon reasonable request.

## Electronic supplementary material

Supplementary video A

Supplementary video B

Supplementary information
