# Supplementary material for: Pinned domain wall oscillator as a tuneable direct current spin wave emitter
Source: Sci Rep. 2017 Oct 19;7:13559. doi: 10.1038/s41598-017-13806-1 (PMC5648888; doi:10.1038/s41598-017-13806-1)
Supplement: Supplementary file 3 — Supplementary information [file 41598_2017_13806_MOESM3_ESM.pdf]

Pinned domain wall oscillator as tunable direct current spin wave emitter
Supplementary information

Michele Voto, Luis Lopez-Diaz, Eduardo Martinez

Departamento de Física Aplicada, Universidad de Salamanca,

Plaza de la Merced s/n. 37008 Salamanca, Spain

SUPPLEMENTARY INFORMATION

A. One dimensional model for pinned DW rotation

In this section we use the one dimensional model[1–3] to derive analytical expressions for the critical currents $J_{\text{rot}}, J_{\text{dep}}$, bounds of the operating window of a pinned DW oscillator.

We start from the one dimensional equations of dynamics in the absence of an external applied field,

$$\dot{q} = \frac{\gamma_0\Delta}{1+\alpha^2}\left(\alpha H_p(q) + \frac{H_K}{2}\sin 2\phi\right) + \frac{1+\alpha\beta}{1+\alpha^2}u \quad (1)$$

$$\dot{\phi} = \frac{\gamma_0}{1+\alpha^2}\left(H_p(q) - \alpha\frac{H_K}{2}\sin 2\phi\right) + \frac{\beta-\alpha}{1+\alpha^2}\frac{u}{\Delta}. \quad (2)$$

The DW will not propagate as long as the pinning restoring force compensates the drive of STT in equation (1). This means that as long as

$$u = u_{\text{dep}} < \frac{\gamma_0\Delta}{2(1+\alpha\beta)}\left(\frac{\alpha k\ell}{\mu_0 M_s L_y L_z}\right)$$

there exists a position $\overline{q}$ such that $\gamma_0\Delta\alpha H_p(\overline{q}) = -(1+\alpha\beta)u$.

Substituting $H_p(\overline{q})$ with this condition in (2) we obtain

$$\dot{\phi} = -\frac{u}{\alpha\Delta} - \frac{\alpha\gamma_0}{(1+\alpha^2)}\frac{H_K}{2}\sin 2\phi. \quad (3)$$

Equilibrium in the system, with no rotation of the DW $\dot{\phi} = 0$, is reached if $\sin 2\phi^* = -\frac{2u}{\gamma_0\Delta H_K}$ which means

$$\phi^* = \frac{1}{2}\arcsin\left(-\frac{2u}{\gamma_0\Delta H_K}\right)$$

only possible if $|u| \le |\frac{\gamma_0\Delta H_K}{2}| = u_{\text{rot}}$.

In the end, we have two bounding conditions for the working window of the DW oscillator $u_{\text{rot}} < u < u_{\text{dep}}$ provided that $u_{\text{rot}} < u_{\text{dep}}$. The parameters playing a role in the extent of the working window are the shape anisotropy H_K which has to be minimized to minimise u_{rot} and the pinning strength k and extent ℓ which have to be maximized to have a large u_{dep}.

On the other hand, if we consider the effect of a uniform external applied field as in the last part of the results section, we can extract the condition for which pinning and external field both along $-z$ equilibrate the STT and, thus, keep the DW pinned for higher applied currents. From

$$\dot{q} = \frac{\gamma_0\Delta}{1+\alpha^2}\left(\alpha\left(H_a + H_p(q)\right) + \frac{H_K}{2}\sin 2\phi\right) + \frac{1+\alpha\beta}{1+\alpha^2}u = 0 \quad (4)$$

we obtain

$$u_D = -\frac{\alpha\gamma_0\Delta}{1+\alpha\beta}\left(H_p(\overline{q}) + H_a\right). \tag{5}$$

Using $u = \frac{J_c P \mu_B}{e M_s}$ we have

$$J_{\text{dep}}(H_a) = \frac{eM_s}{P\mu_B}\frac{\alpha\gamma_0\Delta}{1+\alpha\beta}\left(H_p(\overline{q}) + H_a\right) = J^0_{\text{dep}} + \frac{eM_s}{P\mu_B}\frac{\alpha\gamma_0\Delta}{1+\alpha\beta}H_a, \tag{6}$$

where J^0_{dep} is the threshold depinning current at zero applied field.

B. Non-uniform current density effects on DW dynamics

The current density is expected to spatially vary due to the presence of a constriction along the wire. Its spatial configuration is computed numerically and represented in Fig. S 1 as stream lines.

The spatial variation of current density introduces additional complexity to the problem. In fact, local current density at the DW is maximum when it is located at the centre of the notch and it decreases as the DW is pushed away from the centre, as shown in Fig. S 1. This is clarified by looking at DW rotation frequency and average position as function of the current density measured at the DW position as it is done in Fig. S 2-b. As the current density increases, the DW moves further away from the notch where current density is lower. As can be observed, if we take the data from Fig. S 2-a (same as Fig.4-b in main text) and plot them against the current J_c flowing at the DW position, we obtain at first a linear increase of f_{DW} with J_c as predicted by the analytical model (3). Above 8.5 $\times 10^{10}$ A m^{-2} however, the further displacement of the DW from the centre of the notch and concurrent reduction of the local current density yields a stabilization of the effective current density flowing at the DW, yielding an almost constant f_{DW}. Average DW position is computed from micromagnetic simulations as $\Delta x_{DW} = -\frac{L_c}{2} + \sqrt{\frac{L_c^2}{4} + A_{tot}\langle m_z\rangle}$ representing the height of the trapezoidal region that reversed magnetization assuming the DW as a straight line, as schematically shown in inset in Fig. S 2-a, and A_{tot} is the total upper surface of our sample.

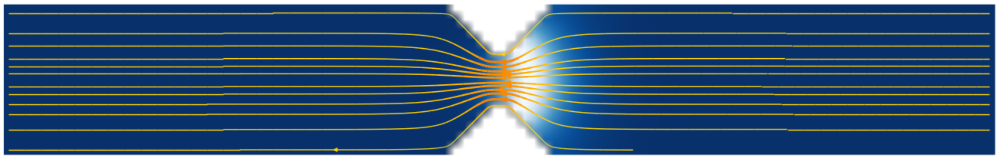

FIG. S 1. Streamplot representing the current density flux at the constriction, the intensity is represented in color scale. A typical DW position during pinned rotation is shown in bright color in the background.

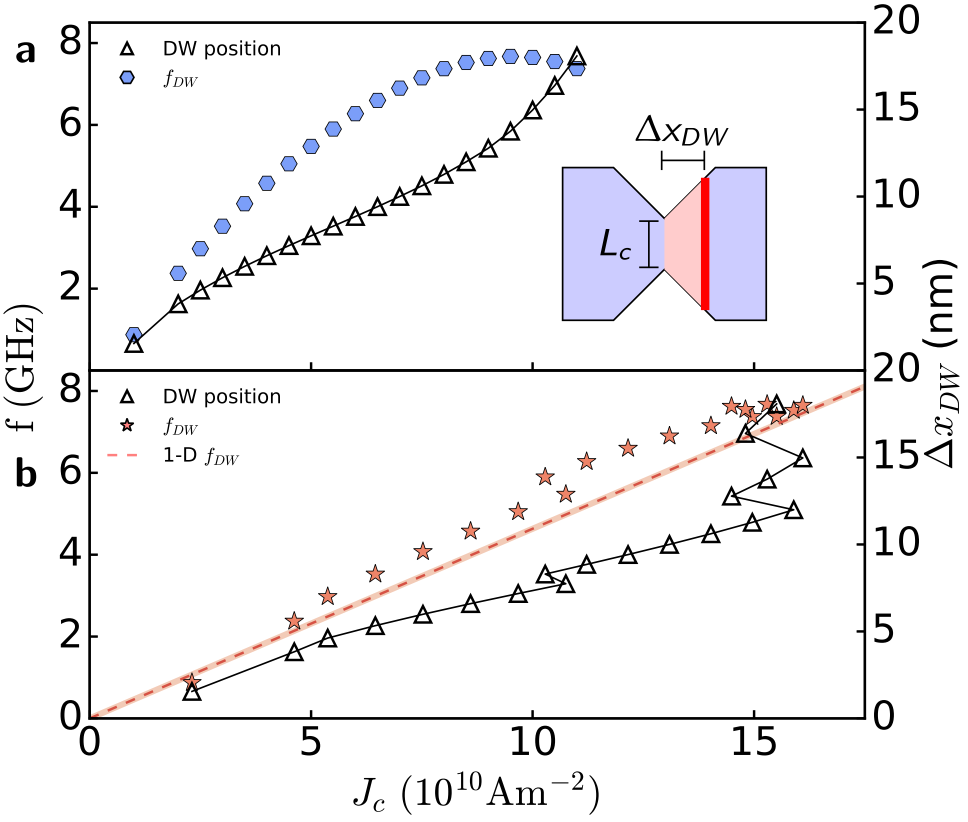

FIG. S 2. **a** DW rotation frequency and position as function of nominal current density. Inset: schematic showing how Δx_{DW} is evaluated. **b** DW rotation frequency and position as function of the current density measured at the centre of the DW. Dashed line is the analytical prediction from equation (3).

---

[1] N. L. Schryer and L. R. Walker, Journal of Applied Physics **45**, 5406 (1974).

[2] A. Mougin, M. Cormier, J. P. Adam, P. J. Metaxas, and J. Ferré, Europhysics Letters (EPL) **78**, 57007 (2007),.

[3] A. Thiaville, Y. Nakatani, J. Miltat, and Y. Suzuki, Europhysics Letters (EPL) **990**, 5 (2004),.
